# Design of a multicentered randomized controlled trial on the clinical and cost effectiveness of schema therapy for personality disorders

**DOI:** 10.1186/1471-2458-12-75

**Published:** 2012-01-24

**Authors:** Lotte LM Bamelis, Silvia MAA Evers, Arnoud Arntz

**Affiliations:** 1 Department of Clinical Psychological Science, Faculty of Psychology, Maastricht University, Maastricht, The Netherlands; 2 Department of Health Services Research, Caphri School of Public Health and Primary Care, Faculty of Health Medicine and Life Sciences, Maastricht University, Maastricht, The Netherlands; 3 Netherlands Institute for Advanced Study in the Humanities and Social Sciences, Royal Netherlands Academy of Arts and Sciences, Wassenaar, the Netherlands

## Abstract

**Background:**

Despite international guidelines describing psychotherapy as first choice for people with personality disorders (PDs), well-designed research on the effectiveness and cost-effectiveness of psychotherapy for PD is scarce. Schema therapy (ST) is a specific form of psychological treatment that proved to be effective for borderline PD. Randomized controlled studies on the effectiveness of ST for other PDs are lacking. Another not yet tested new specialized treatment is Clarification Oriented Psychotherapy (COP). The aim of this project is to perform an effectiveness study as well as an economic evaluation study (cost effectiveness as well as cost-utility) comparing ST versus COP versus treatment as usual (TAU). In this study, we focus on avoidant, dependent, obsessive-compulsive, paranoid, histrionic and narcissistic PD.

**Methods/Design:**

In a multicentered randomized controlled trial, ST, and COP as an extra experimental condition, are compared to TAU. Minimal 300 patients are recruited in 12 mental health institutes throughout the Netherlands, and receive an extensive screening prior to enrolment in the study. When eligible, they are randomly assigned to one of the intervention groups. An economic evaluation and a qualitative research study on patient and therapist perspectives on ST are embedded in this trial. Outcome assessments (both for clinical effectiveness and economic evaluation) take place at 6,12,18,24 and 36 months after start of treatment. Primary outcome is recovery from PD; secondary measures include general psychopathological complaints, social functioning and quality of life. Data for the cost-effectiveness and cost-utility analyses are collected by using a retrospective cost interview. Information on patient and therapist perspectives is gathered using in-depth interviews and focus groups, and focuses on possible helpful and impeding aspects of ST.

**Discussion:**

This trial is the first to compare ST and COP head-to-head with TAU for people with a cluster C, paranoid, histrionic and/or narcissistic PD. By combining clinical effectiveness data with an economic evaluation and with direct information from primary stakeholders, this trial offers a complete and thorough view on ST as a contribution to the improvement of treatment for this PD patient group.

**Trial registration:**

Netherlands Trial Register (NTR): NTR566

## Background

Personality disorders (PDs) are characterized by an enduring, pervasive and pathological pattern of thoughts, feeling and behavior expressed in a dysfunctional and inappropriate manner, which is deviant from societal norms. In the DSM-IV, personality disorders are grouped into 3 clusters: the 'odd, eccentric' cluster A (paranoid, schizotypal and schizoid PD), the 'dramatic' cluster B (borderline, antisocial, histrionic and narcissistic PD), and the 'anxious' cluster C (avoidant, dependent and obsessive-compulsive PD) [1]. Despite the frequent application of prolonged psychotherapy for people with personality disorders, controlled research into the clinical and cost-effectiveness of psychotherapy is scarce [2]. This is remarkable, given the substantial burden on patients and society. Moreover, PDs are highly prevalent, as seen in numbers ranging from 3 to 15% in community population up to as high as 80-90% in secondary health care settings [3-6]. PD-patients show chronic dysfunctions on social and interpersonal level [7-10] and experience substantial impairment in work and basic self-care [11], leading to an enormous negative impact on the patient's life and the life of his/her close relatives. Furthermore, PDs and personality-related factors play a key role in the development and progress of other mental disorders, as demonstrated in numerous cross-sectional and prospective studies [12-16].

Apart from patients, also society bears the costs of chronic personality pathology. Factors attributing to these notable costs are increased health care utilization, productivity losses, and unstable employment throughout the lifespan [17,18]. A recent study showed that the costs of untreated PDs in the Netherlands are substantially higher than those of other psychopathology like depression or generalized anxiety disorder [19].

The evidence on treatment effectiveness for PDs that exists so far is mostly restricted to borderline PD (BPD) [20]. Several reviews report large effect sizes of specialized psychological treatments for all PDs in general [21] and specifically for cluster-C [22]. However, it should be noted that most studies into treatment of non-BPD PDs are of questionable methodological quality and show conflicting results. A few RCTs focused on cluster-C PDs. Emmelkamp et al. [23] showed greater improvement in cognitive over psychodynamic psychotherapy for Avoidant PD, while Svartberg, Stiles & Seltzer [24] found these treatment forms to be equally effective for Cluster C PDs. Another study [25] comparing manualized versus non-manualized dynamic psychotherapy showed equal decreases in the severity of PD symptoms, but both treatment conditions failed to reduce psychiatric symptoms to a 'healthy' level at post-test. Inconsistent findings also appear when focusing on different treatment modalities instead of different theoretical frameworks for cluster C. Recent evidence suggests that outpatient psychotherapy is equally effective as day treatment [26], while a naturalistic study [27] showed only modest improvement for outpatient therapy following day treatment in which considerable progress was made. Bartak et al. [28] compared different treatment modalities for cluster C in a multicenter non-experimental study in the Netherlands, and results favored short-term inpatient treatment over other treatment modalities. On top of these contradictory findings, studies on treatment effectiveness involving cluster C are often difficult to interpret because this group of PDs is mostly not the main research focus but partly allowed as comorbid psychopathology (e.g. [29,30]). Methodologically sound scientific investigation of treatment effectiveness for paranoid, histrionic and narcissistic PD hardly exists [31-33].

In a budget-constrained society, other important aspects in the evaluation of a new treatment form are costs and benefits of treatment. Unfortunately, for this patient group the same paucity of controlled cost-effectiveness studies is seen as with clinical effectiveness. The few studies that suggest cost-effectiveness often are not based on formal and well-prepared cost analyses [34]. An exception herein is an economic evaluation study alongside the Dutch non-experimental study mentioned before, in which cost-effectiveness of different treatment modalities for both cluster B and C PDs is assessed [35,36]. Results show that optimal treatment choice depends on what threshold is considered acceptable. Although this research group executed pioneering research in economic evaluations for non-BPD patient groups, findings are difficult to interpret because of different focus (modalities instead of theoretical framework) and non-randomization of patients.

Schema therapy (ST) gained a lot of attention the past decade as a promising treatment for PDs. Clinical effectiveness is shown for borderline PD both in an extensive RCT (comparing ST head-to-head with Transference Focused Psychotherapy (TFP) [37]) and in a Dutch implementation study [38]. Also in group format ST showed positive results for borderline PD [39]. The first RCT mentioned demonstrated ST to be less costly and more effective than TFP, so preferable in terms of cost-effectiveness [40].

Because the aforementioned shortcomings, the need for properly designed studies of psychological interventions for non-Borderline PDs is pressing [33,41]. This is especially important as psychological treatment is considered to be the treatment of choice for these disorders [34]. Despite some evidence for the effectiveness of ST techniques for PDs other than borderline [42], properly designed effectiveness and economic evaluation studies comparing ST with other psychological treatments for non-borderline PDs are lacking. The main objective of this study is to evaluate the clinical and cost effectiveness of ST for a group of 6 PDs: avoidant, dependent, obsessive-compulsive, paranoid, histrionic and narcissistic PDs. Other PDs (borderline, antisocial, schizotypal and schizoid) are excluded as they are deemed to require highly specialized treatment protocols and higher dosage of treatment. In this study a treatment protocol of 50 ST-sessions is compared to treatment as usual (TAU). To assess to what degree a possible positive effect of ST is the result of the effects of a new specialized and promising treatment, we add the comparison of TAU with another specialized treatment, clarification oriented psychotherapy (COP), a form of client centered therapy developed for PDs [43], to the design. Apart from the clinical effect study and economic analysis, in a qualitative research part patients and therapists are asked to provide insight in helpful and not helpful aspects of the ST protocol. By collecting input from direct users, valuable information is obtained to improve the ST protocol and tailor it to the needs of primary stakeholders.

The following research questions are defined:

Effectiveness study

How do ST and COP compare to TAU, in terms of recovery from PD-diagnosis, reduction of psychopathological symptoms and improvement of quality of life?

Are these new treatments better in retaining patients in therapy than TAU?

Economic evaluation

From a societal perspective, are ST and COP preferable to TAU in terms of costs, effects and utilities?

Patient and therapist perspectives

What do patients and therapists believe to be helpful and not helpful factors in the ST protocol?

Based on the superiority of ST found in previous research, we hypothesize that (a) ST shows greater clinical improvement than TAU, and (b) seen from a societal perspective, ST is more cost effective in terms of costs and utilities. Similarly, we test whether another specialized treatment of PD, COP, is superior to TAU in these respects.

## Methods

This trial includes a clinical effectiveness study, an economic evaluation and a qualitative research part regarding patient and therapist perspectives on ST.

### Clinical effect study

#### Design

**Figure 1:**
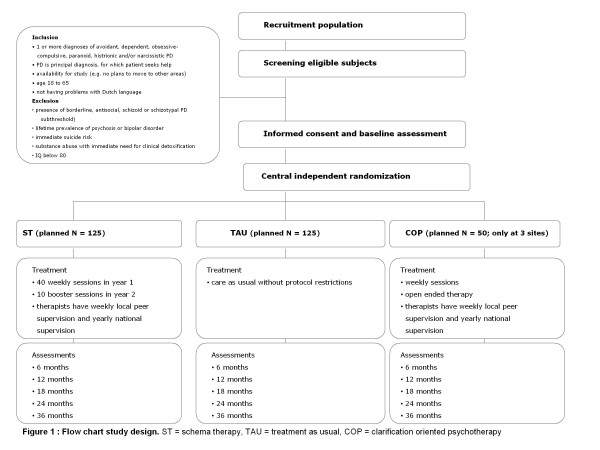
**Flow chart study design**. ST = schema therapy, TAU = treatment as usual, COP = clarification oriented psychotherapy.

The study is a multicentered randomized controlled trial (RCT), in which patients are assigned to either ST or TAU, while in 3 centers COP is added as a third treatment condition. In Figure 1, information on patient flow, screening procedures and intermittent assessments is graphically shown. The research protocol is approved by the Medical Ethical Committee of Maastricht University/University Hospital Maastricht and by local committees in participating centers.

#### Recruitment

Patients are recruited in 12 mental health care institutes throughout the Netherlands (Geestgronden Noord-Holland, GGZ Oost-Brabant, GGZ Nijmegen, Mediant Enschede, Mondriaan Zorggroep Heerlen, Reinier van Arkelgroep Den Bosch, Riagg Maastricht, Riagg Rijnmond, Riagg Zuid Roermond, Rivierduinen Leiden, Symfora Hilversum, and UMC Radboud Nijmegen). They are referred to the study either during intake (i.e. at first contact with mental health care institute), after having received previous care that failed to reduce their PD problems, or by clinicians treating these patients for chronic problems. After patients receive both written and oral information and signed informed consent, in- and exclusion criteria are checked.

#### Participants

Patients are eligible if they have one or more diagnoses of avoidant, dependent, obsessive-compulsive, paranoid, histrionic and/or narcissistic PD. This PD has to be the principal diagnosis, according to both patient and clinical staff, and patients should request help for PD-related problems. Further, inclusion criteria are age 18-65 and not having problems with Dutch language (talking, understanding, reading and writing). In- and exclusion criteria are described in Figure 1.

At baseline clinical diagnoses are assessed with the Dutch version of the Structured Clinical Interviews (SCID) for DSM-IV Axis-I and Axis-II disorders ([44-47] and executed by extensively trained clinicians at local sites). For clients having more than 1 PD diagnosis, main diagnosis is determined by the interviewer or -if missing- by 2 raters (LB & AA) after clinical judgment and taking into account self-reported reason for seeking help.

#### Sample size

Power calculations for ST and TAU are based on the results from the BPD trial comparing ST and Transference Focused Psychotherapy (TFP) [37]. The study is powered to detect a difference between ST and TAU in proportion recovered patients of 46 vs. 24% (OR = 2.70). With alpha = .05 (2-tailed), n = 100 per condition yields a power of 90%. To account for possible dropouts (ca. 18% expected based on [48]), 125 patients are needed per condition. With the planned sample sizes (ST = 125; TAU = 125; COP = 50) power is 78% (70% after dropout) to detect a similar difference between COP and TAU.

#### Randomization and procedure

Following inclusion in this trial, patients complete an extensive baseline assessment prior to randomization. An independent statistician generated a computerized randomization list using Adaptive Biased Urn Randomization for small strata [49], which keeps randomization unpredictable up to and including the last patient on each site, while keeping the group sizes at each site in good balance. A study-independent central research assistant checks in/exclusion criteria, and reads the next available treatment condition per center from this randomization list, and passes it on to local staff by e-mail. Once allocated, further matching between patient and therapist is allowed and performed by local teams (ST & COP) and regular intake staff (TAU).

After start of treatment, assessments occur every 6 months during the first 2 years, followed by a follow up assessment 3 years after start of therapy. All assessments are executed by independent research assistants at local sites, except for SCID-interviews which are performed by trained clinicians at local sites during inclusion period and by trained independent interviewers at follow-up. In general, assessments comprise self-report questionnaires (administered on PC), a cost-interview and a computer task. When study dropout is imminent due to unwillingness to come to the center for assessments, questionnaires could be filled out at home. In these cases, cost interviews are administered by telephone. Given the nature of our study, blinding of participants and research assistants is not possible. However, the primary outcome is assessed by independent central interviewers, blind for condition. To optimize similarity in assessments, research assistants receive a one-day training regarding the study protocol given by members of the central research committee of Maastricht University, discuss process in regular telephonic meetings and use an extensive standardized study protocol.

#### Treatments

All sites offer ST and TAU. In 3 centers a third treatment condition (COP) is added. In this way, a standardized and highly specialized outpatient treatment is added as an extra control. Because of restricted COP-therapist capacity at most sites, COP is only offered at 3 sites.

ST and COP are characterized by the following commonalities: therapists receive extensive expert-training at study start, yearly national supervision and weekly peer-supervision at local sites; both are outpatient treatments, (initially) delivered in weekly sessions; additional psychological treatment with focus on personality pathology is not allowed; and psychopharmacological treatment is permitted, but only on clinical indication according to an independent psychiatrist.

##### Schema therapy (ST)

Schema therapy (ST) was originally developed for people with severe personality pathology and combines experiential, cognitive-behavioral, psychodynamic and interpersonal techniques [50]. The concept of schema modes is central in current ST [51]. ST aims at reducing maladaptive modes and strengthening the healthy adult mode. Arntz & Young created a theoretical model containing the most prevalent modes for the 6 PDs under study [52,53]. For this study, a 50-session treatment protocol is developed with 40 weekly sessions in year 1 and 10 booster sessions in year 2. The treatment protocol is naturalistic in the sense that, apart from the framework directing treatment focus throughout sessions, therapists do not have a session-to session detailed treatment manual.

At start of treatment, a maximum of 6 sessions is spent on introduction, patient history and case conceptualization. During the first half year, focus mainly lies on in-session child, parent and coping modes with emphasis on processing childhood experiences through imagery rescripting and other experiential techniques. In the second half year, current life experiences and active changing of behavior are targeted. Booster sessions in the second year could be spread according to the wishes of therapist and patient, and are intended to maintain newly acquainted healthy behavior, recognize possible pitfalls and prevent relapse.

##### Clarification oriented psychotherapy (COP)

COP is a treatment rooted in client centered therapy (CCT), developed by Sachse [43,54]. It was designed to clarify and restructure dysfunctional cognitive-affective schemas focusing strongly on interpersonal behavior and problems. COP also offers specific techniques in the treatment of PDs. In this model, PDs are conceptualized on a dimension ranging from slight characteristics of a PD (not diagnosed by DSM-IV), to pathological and extreme interaction patterns. Based on unmet social needs in childhood or youth, interaction patterns are developed which may have been functional at the time, but are not anymore. According to Sachse, PD-characteristics occur at 3 levels: (1) the level of authentic needs: appreciation, importance for (to) others, reliability of relationships, solidarity of relationships, autonomy and boundaries (2) the level of schemas, which are assumptions about the self and others, and (3) the level of play, which is highly automated and not transparent, manipulative and controlling. Whilst this play behavior is intended to get needs fulfilled, this often doesn't work. The goal of treatment is to enlarge the authentic being of patients, by satisfying authentic needs, gaining insight in schemas and reducing play behavior. Therapeutic techniques to achieve this are being complementary with authentic needs, uncomplimentary with play behavior, confronting with play behavior and clarifying and restructuring rigid schemas. Just like the first year in the ST protocol, COP is offered weekly to patients. Contrary to the ST protocol, COP is open ended without a strict number of treatment sessions, as this fits best with its CCT nature.

##### Treatment as usual (TAU)

In this treatment condition, treatment is whatever care (except ST/COP) a patient would receive if the study would not take place. Generally TAU is expected to follow the multidisciplinary clinical guidelines for PDs in the Netherlands [55]. When allocated to TAU condition, the regular intake staffs at local sites indicate the specific treatment format for that patient. Thus, the matching of patient to type of regular treatment by the responsible clinicians at the site is part of TAU. In this way, TAU is optimized and mimics usual practice.

#### Therapists, training, and treatment integrity check

ST and COP therapists received an extensive 4 day expert-training before study start. Due to one center withdrawing participation after training but before recruiting patients, two extra sites were added to compensate, and extra therapists (also from the first group of centers) were trained in ST in a second training. This creates two 'waves' in the study (from now on referred to as therapy cohorts). There are potentially important differences between the two ST-trainings. The first cohort was trained in a foreign language (English) and consisted of 73 therapists, while the second cohort consisted of 20 therapists who were trained in their native language (Dutch). Training in the first cohort consisted mainly of lectures and video demonstrations, whereas the second training was much more structured with short instructions, life demonstration, and compulsory role-plays for the participants to train the main therapeutic techniques.

Therapists are uniquely assigned to one of the treatment conditions to prevent contamination, with the exception of 6 ST and 3 COP therapists who also act as a group-therapist in TAU. This exception is allowed as these therapists have non-ST/COP co-therapists that help them to stick to the non-ST/COP format of these group therapies.

Adherence to treatment protocol and absence of techniques and elements from contrasting treatments is checked. Except for group treatment in TAU (where non-study participants do not give consent to record sessions), all treatment sessions are audio taped. Eventually, 3 sessions per patient are randomly selected for evaluation; 1 from the first 5 weeks, 1 from the first half year and 1 from the second half year of treatment. An instrument to measure treatment adherence is developed based on the ST Therapy Adherence and Competence Scale for BPD [56], the Collaborative Study Psychotherapy Rating Scale 6 (CSPRS-6);[57,58], and elaborated consultation with ST- and COP-experts.

### Economic evaluation study

The economic evaluation is performed from a societal perspective, and involves a combination of a cost-effectiveness analysis (CEA) and a cost-utility analysis (CUA). In a CEA effects are presented in clinical outcomes (in our study recovery of diagnosis). The primary outcome measures for the cost-utility analysis are QALYs, based on the EuroQol utility scores [59,60]. Comparing ST and COP separately with TAU yields four Incremental Cost Effectiveness Ratios (ICERs) that express the incremental costs per recovered patient and per QALY gained. The net monetary benefit (NMB) is feasible to compare three treatments in one analysis. The NMB is calculated by multiplying the increase in effectiveness by the amount decision-makers are willing to pay for one extra unit of effect, minus the increase in costs. In the base-case analyses, a monetary threshold of € 20,000 is used [40].

For the identification of costs, a division is made into healthcare costs and productivity losses. Because it is very hard to make a clear distinction between PD-related and non-PD-related costs, all costs are taken into account. Costs are divided into (1) main intervention costs in participating health care center (i.e. treatment patients were randomly assigned to) (2) other mental health care received, (3) health care costs, containing medication (divided in prescribed and over the counter medication), general practitioner, emergency care, outpatient consults in general hospital, and admissions to general hospital, and (4) productivity costs (further divided in costs due to PDs and costs due to other complaints).

### Patient and therapist perspectives

We aim to identify specific helpful and not helpful aspects of the ST protocol used in this study. This information is derived by using qualitative research methods with primary stakeholders (patients and therapists). A selection of ST patients and therapists is asked to participate in this qualitative study, as many as necessary until saturation appears (when no more information is added and replication occurs). Patients receive semi structured in-depth interviews in the early phases of treatment (time point 1) and after completion of treatment (time point 2). At time point 2, also patients who dropped out of treatment are interviewed. Therapists share their experiences in a focus group (structured group session in which thoughts and views about certain predefined ST topics are exchanged, led by a chairperson) at equal time points. Some main topics are: helpful and harmful aspects of ST, the use of specific ST techniques, therapeutic relationship, supervision and training of therapists.

Patient interviews and therapist focus groups are recorded and fully transcribed. All participants receive a verbatim transcript and are asked to verify whether their opinion is expressed correctly (member check).

#### Instruments

Instruments are used in the screening process, the clinical effect study and the economic evaluation study. In Table 1 an overview of all instruments per time point is shown.

**Table 1:** Overview of instruments per time point

	Screening	Baseline	6 months	12 months	18 months	24 months	36 months
SCID II	**•**						**•**
SCID I	**•**						**•**
ADP-IV		**•**	**•**	**•**	**•**	**•**	**•**
GAF		**•**	**•**	**•**	**•**	**•**	**•**
SOFAS		**•**	**•**	**•**	**•**	**•**	**•**
SCL-90		**•**	**•**	**•**	**•**	**•**	**•**
WSAS		**•**	**•**	**•**	**•**	**•**	**•**
MSGO		**•**	**•**	**•**	**•**	**•**	**•**
Whoqol-short version		**•**	**•**	**•**	**•**	**•**	**•**
Euroqol-5D		**•**	**•**	**•**	**•**	**•**	**•**
Cost interview		**•**	**•**	**•**	**•**	**•**	**•**

SCID I = Structured Clinical Interview for Axis-I Disorders, SCID-II = Structured Clinical Interview for Axis-II Disorders, ADP-IV = Assessment of DSM-IV Personality Disorders Questionnaire, GAF = Global Assessment of Functioning, SOFAS = Social and Occupational Functioning Scale, SCL-90 = Symptom Check List, WSAS = Work and Social Adjustment Scale, MSGO = Miskimins Self Goal Other, Whoqol-short version = World Health Organization Quality of Life Questionnaire - short version

### Primary outcome and PD-diagnosis

Structured clinical interview for personality disorders (SCID-II)

Primary outcome is presence versus absence of PDs, which is assessed with SCID-II-interviews at follow-up 3 years after start of treatment. Interviews are administered by telephone after the follow up assessment at local sites and executed by a group of independent raters blinded for condition. Raters are trained in SCID interviews, and inter rater reliability is assessed. Only those PD-modules and criteria are assessed on which patients scored at baseline and further all specific DSM-IV criteria on which patient scored 4 or more on the 1–7 scale of the ADP-IV at follow up (when 3 or more criteria per PD had a >4 score, the complete PD had to be assessed). Each relevant PD-criterion has to be scored as absent (score 1), questionable (score 2), or present (score 3) for both the 6 months prior to FU, and the half year before that. The sum of 3-scores points out whether sufficient criteria are met for a specific PD-diagnosis. Previous studies found adequate to good inter-rater reliability for SCID II interviews [61][62][63]. Recovery from diagnosis as measured with SCID II is the primary outcome measure in this study. When patients do not exceed the minimal number of criteria needed to obtain a diagnosis on any of the 6 PDs under study, they are considered to be recovered. Because of the lenience of this criterion, we assess sensitivity by reanalyzing presence vs. absence of subthreshold diagnoses (defined by meeting one PD-criterion less than needed for a full diagnosis). 

### Secondary outcomes

Assessment of DSM-IV personality disorders questionnaire (ADP-IV)

At every intermediate and follow up assessment, PD-pathology and associated distress are assessed with the ADP-IV [64]. With this self-report questionnaire, DSM-IV PD criteria are assessed. Patients have to indicate on a 7-point Likert scale to what degree PD criteria hold for them, ranging from 1 ('not at all') to 7 ('completely'), and whether they experience distress from it (on a range from 1-not at all to 3-definitely). Item construction of the ADP-IV allows for both dimensional and categorical diagnostic evaluation [65]. Adequate internal consistency, validity and reliability were shown consistently in previous studies [65-67].

Structured clinical interview for axis I disorders (SCID I)

SCID I is used both as screening and outcome instrument. During screening, SCID I [44,46] is used to check for Axis I diagnoses that might lead to exclusion (psychosis, bipolar disorder, substance abuse). At follow up, Axis-I diagnoses of mood and anxiety disorders (as the most prevalent at baseline) are assessed by the same blind interviewers registering SCID II. SCID I proved to have acceptable psychometric properties [61].

Global assessment of functioning scale (GAF) and social and occupational functioning scale (SOFAS)

These 100-point scales are derived from DSM Axis V, to assess global functioning & symptom severity (GAF) and social functioning (SOFAS). Both scales are administered by the research assistant at each measurement after a semi-structured interview to elicit relevant information. Lower scores indicate poorer functioning and greater symptom severity. These scales have shown to be a valid and reliable rating scale of global psychopathology [68,69].

Symptom check list (SCL-90)

General psychopathological symptoms are assessed with the SCL-90 [70]. This self-report inventory contains 90 items that have to be scored on a 5-point Likert scale of distress, ranging from 'not at all' to 'extremely'. The total score can be used as a measure of global symptom severity. The SCL-90 has good psychometric properties [71].

Work and social adjustment scale (WSAS)

The WSAS is a simple 5-item list used to assess general impairment on several life domains like work, household, social and private leisure, family and relationships [72]. Each item has to be scored on a range from 0 to 8. Higher scores denote more disability. This instrument proved to be reliable, valid and change-sensitive in various patient populations [73].

Miskimins self goal other (MSGO)

The MSGO is a measure to assess discrepancy between actual and ideal self-perception [74]. 30 personality trait dimensions have to be scored on a 100 mm visual analogue scale, for current and ideal self-perception respectively. Discrepancy scores are derived by calculating the mean difference across items. The MSGO has shown adequate psychometric properties in previous studies [42,75].

World health organisation quality of life questionnaire (Whoqol-short)

Quality of life is assessed with a modified version of the World Health Organisation Quality of Life Questionnaire. Patients have to self-report to what extent they experience quality of life on several domains (physical, psychological, social relationships, environment, positive feelings, negative feelings, self-esteem) [76,77]. Psychometric studies revealed the WHOQOL to be a valid and reliable measure.

Euroqol-5D

The EuroQol is a standardized non-disease specific instrument for describing and valuing health-related quality of life. Next to the assessment of quality of life on five health-state dimensions (mobility, self-care, usual activity, pain/discomfort and anxiety/depression), the EuroQol thermometer ranges someone's current health status between 0 and 100 [59]. For the cost-utility analysis, the profiles resulting from the five health-state dimensions can be converted into utilities based on the social tariffs of the EuroQol, the so-called EQ-5D UK value set [78]. Utilities refer to the preference for any particular set of health outcomes and are generally indicated by a number between 0 and 1. Utilities at different time points are used to compute QALYs. A QALY combines preferences for both length of survival and its quality into one single measure.

Resource use

For the resources, a distinction is made between intervention costs and other resource use. The number of sessions and specific content of interventions are gathered by local research assistants, while costs of all other resources are measured by means of a structured cost interview [79] at every assessment. In this interview, patients are asked to specify work status and absenteeism, and they report the use of medication, GP visits and contacts with different services. The cost interview is retrospective in nature. For most questions a 3-month recall period is used, except for costly admissions to general or psychiatric hospitals and crisis services, in which a 6 or 12 month recall period is used (for intermittent and follow up assessments respectively).

### Analyses

All analyses are planned to be carried out with SPSS 19.0 and MLWin 1.10, while bootstrap simulations are done using Excel.

#### Clinical analyses

Data analyses are based on intention to treat analyses (including all patients regardless of whether they drop out from treatment or not), using all available data. Intermittent missing data on item level are imputed by mean values from previous and subsequent time points.

Primary outcome (recovery from PD diagnosis) and dichotomous secondary outcome parameters (absence of comorbid depression or anxiety disorder), are analyzed with multilevel logistic regression. Continuous secondary outcome parameters are analyzed with multilevel mixed models. In these multilevel models center is treated as a random factor (i.e., random intercepts and slopes (if applicable)). If changes over time are mainly linear, linear trend in time is inspected; otherwise the time variable is transformed to obtain a linear relationship. When outcome parameters are not distributed normally, appropriate transformations are used.

Predictors of outcome are incorporated in the analyses. To control for baseline severity a composite measure is constructed out of standardized baseline values of: # axis-I disorders, # axis-II disorders, ADP-4 trait sum score, ADP-4 distress sum score, SCL-90, GAF, SOFAS, disability status (biographical variable). Being new vs. 'chronic' patient is not associated with these variables, neither with outcome, thus left out of consideration. The severity-index is used as covariate in outcome analyses. Since the difference between a more passive and a more active, experiential-learning oriented training is potentially very important for implementation and for effectiveness of treatment, we include this variable in the analyses to test for moderation, and to control for possible effects. For 2 sites, all ST therapists were trained in the second cohort; therefore all TAU-patients of these sites are their patients' controls. For other sites, a minority of ST-therapists was trained in the second cohort, and control TAU patients are selected per site by matching TAU-patients to the 2nd cohort ST-therapists' patients on the basis of 1st diagnosis, gender and age.

Given the strong positive effect of ST on treatment retention found in earlier studies [37-39], dropout is analyzed with both multilevel logistic regression and survival analysis (to account for development over time).

To assess treatment integrity, a random selection of audiotapes of treatment sessions is rated by trained independent judges, blind for condition. A subset of recordings is rerated to estimate inter rater reliability, expressed with the intra class correlation coefficient (ICC).

#### Economic evaluation

The economic analysis is also performed according to the intention-to-treat principle. As mentioned before, both a cost-effectiveness analysis CEA (expressing effects as recovery of diagnosis) and cost-utility analysis CUA (effects expressed as QALYs) are done.

For the valuation of cost prices, standard Dutch unit prices are used [80] or-when unavailable- average tariffs. Prescribed medication costs are based on the Dutch Pharmacotherapeutic Compass [81]. Productivity costs are calculated according to the human capital method (total productivity costs are the product of total hours lost with hourly wage), as this method is preferable in patient groups with apparent disease-related work disability [82].

Costs are calculated by multiplying volumes with price per cost item. All prices are expressed in Euros for the year 2007 (since the majority of treatments started in this year). If necessary, costs are indexed to the year 2007 by means of the consumer price indexes of the Dutch Central Bureau of Statistics (CBS). Since the time horizon of the study is 3 years, a discount of 4% per year is applied.

Differences between experimental and control groups in quality of life are analyzed with ANOVA or non-parametric alternatives at the *p *< .05 significance level. Because of the usually non-normal distribution of costs, bootstrapping is used to calculate 95% confidence intervals around costs.

For base-case analyses, primary outcome parameters are proportion of recovered patients (CEA), and total QALY gained during 3 years (CUA).

Uncertainty around costs and effects is dealt with by performing several sensitivity analyses following base-case analyses. Some sensitivity analyses are: analyzing only data from study completers (patients who have complete data sets at every intermittent assessment as well as follow up), correcting for baseline costs and utilities, using Dutch [83] instead of UK value set for QALYs.

Bootstrap simulations are used to estimate sample uncertainty around the cost-effectiveness ratios and are plotted in cost effectiveness planes, in which the position of the bootstrapped cost-effectiveness pairs gives an indication for possible superiority of one treatment over another. In a cost effectiveness acceptability curve (CEAC), the probability of ST being superior across a range of willingness to pay thresholds is plotted. Since the maximum amount of money that society wants to pay is unknown, the monetary threshold per QALY will be varied between € 0 and € 80,000 [84].

#### Patient and therapists perspectives

Content analyses are executed on the verbatim transcripts produced by interviews and focus groups. Recurring themes and topics are labeled and clustered.

## Discussion

Personality disorders (PDs) are complex mental health problems associated with low levels of quality of life, high health care and general society costs, and poor prognosis. Psychological treatment is considered to be the treatment of choice, but research into the clinical and cost effectiveness is sparse and strongly focused on borderline PD. The current study aims to study the effectiveness of schema therapy (ST) compared to treatment as usual (TAU) for 6 PDs not so often studied: cluster-C (i.e. avoidant, dependent, obsessive-compulsive), paranoid, histrionic and narcissistic PD. As an additional control another specialized treatment, clarification oriented psychotherapy (COP), is added as a third condition in 3 of the 12 participating centers.

### Methodological considerations

For our study, a large group of patients and cooperation with many different mental health institutes is necessary. The large number of patients and centers included makes generalizability to population level credible. Following a group of PD patients for a 3 year period in an RCT design is unique in the field. Recommendations for psychotherapy research for personality disorders [48] are taken into account, such as: (a) long duration of treatment and follow up measurement, giving the opportunity to study core characterological change rather than symptomatic change, (b) use of psychometrically sound and well-known outcome measures, and (c) naturalistic ST and COP protocols, resembling real clinical practice better than tight and detailed manuals.

Several limitations and possible pitfalls should be noted. First, due to time restrictions on the inclusion period, the diagnostic profile of patients is mainly determined by the natural flow of patients at local sites. A possibly uneven distribution between the 6 PDs under study is a consequence of that, which might limit the possibilities to draw conclusions on PDs with small numbers. Second, comparing an experimental treatment condition with treatment as usual sets boundaries to the possibility to control for various potentially influencing factors (e.g. uneven duration of treatment, frequency of sessions, etc.). We try to overcome this by adding a second experimental condition (COP). The choice for TAU has merits and demerits. For relatively new treatments such as ST a comparison to treatment as usual is a valuable first step: one would at least require that a new treatment excels existing practice. TAU includes in our study potentially many different treatments, which reduces the kind of control experimental psychologists would like to see (e.g., of factors like attention, frequency, expectations, etc.). On the other hand, the external validity of the control condition is increased, and TAU is optimized by having clinicians making decisions on what type of regular treatment to offer (e.g., see [85] for evidence that intakers can predict effectiveness of psychodynamic therapy). Moreover, for cost-effectiveness the comparison of a new treatment to usual practice is the gold standard [86].

Another important problem potentially affecting the quality of the study is created by the organizational scale of a trial in which so many parties (sites, patients, therapists, coordinators, and research assistants) are involved. The study's scale and the limited financial resources preclude the possibilities for continuous and close monitoring and steering. During the trial, researchers and centers need the capacity and flexibility to overcome numerous problems, e.g. insufficient inclusion, loss of participating centers/research assistants/therapists and the enrolment of new ones, inadequate execution of study protocol, etc. The large scale of the study, at least for psychotherapy research, reduces possibilities to intensively supervise and control treatment delivery. In that sense, the present study is a true effectiveness study, differing from highly controlled efficacy studies.

As to the assessments, we choose for independent and blinded interviewers for assessing the primary outcome. Whilst this is a strong point, financial and logistic limitations preclude that secondary interview outcomes and the cost-interview are taken by blinded research assistants. Lastly, the primary outcome is defined by absence of PD at 3-yr follow-up, operationalized by not meeting criteria for a PD on the SCID-II. This is a bit lenient criterion, and therefore we assess sensitivity of results by a reanalysis with a stricter criterion, that is the absence of full and subthreshold PDs.

## Conclusion

Schema therapy is gaining interest worldwide as a treatment for personality disorders. However, its effectiveness for most personality disorders is so far unknown. This study gives a unique opportunity to fill this gap of knowledge by combining clinical and cost effectiveness analyses within a large group of PD patients.

## Abbreviations

ADP-IV: Assessment of DSM-IV Personality Disorders Questionnaire; ANOVA: Analysis of variance; BPD: Borderline personality disorder; CBS: Central Bureau of Statistics; CCT: Client centered therapy; CEA: Cost effectiveness analysis; CEAC: Cost effectiveness acceptability curve; COP: Clarification oriented psychotherapy; CUA: Cost utility analysis; DSM-IV: Diagnostic Statistical Manual 4th edition; GAF: Global Assessment of Functioning; GP: General practitioner; ICC: Intra class correlation coefficient; MSGO: Miskimins Self Goal Other; NMB: Net monetary benefit; PD: Personality disorder; QALY: Quality Adjusted Life Years; RCT: Randomized controlled trial; SCID: Structured clinical interview for DSM-IV Disorders; SCL-90: Symptom Check List; SOFAS: Social and Occupational Functioning Scale; ST: Schema therapy; TAU: Treatment as usual; TFP: transference-focused psychotherapy; WSAS: Work and Social Adjustment Scale; WHOQOL: World Health Organisation Quality of Life Questionnaire.

## Competing interests

The authors declare that they have no competing interests.

## Authors' contributions

All authors participated in the design of the study. AA obtained funding for this study. All authors drafted, read and approved the final manuscript.

## Pre-publication history

The pre-publication history for this paper can be accessed here:

http://www.biomedcentral.com/1471-2458/12/75/prepub
